# Digital Health Literacy in the General Population: National Cross-Sectional Survey Study

**DOI:** 10.2196/67780

**Published:** 2025-09-22

**Authors:** Junghee Yoon, Seongwoo Yang, Soo Jin Kang, Mangyeong Lee, Dokyoon Kim, Joungwon Park, Su Jin Kim, Jiyoon Han, Jungin Joo, Juhee Cho

**Affiliations:** 1 Department of Clinical Research Design and Evaluation, SAIHST, Sungkyunkwan University, 115 Irwon-ro, Gangnam-gu, Seoul, 06351, Republic of Korea, 82 2-3410-1448, 82 2-3410-6639; 2 Institute for Quality of Life in Cancer, Samsung Medical Center, Seoul, Republic of Korea; 3 Center for Clinical Epidemiology, Samsung Medical Center, Sungkyunkwan University School of Medicine, Seoul, Republic of Korea; 4 Department of Digital Health, SAIHST, Sungkyunkwan University, Seoul, Republic of Korea; 5 Department of Nursing, Daegu University, Daegu, Republic of Korea; 6 AI Research Center, Research Institute for Future Medicine, Samsung Medical Center, Seoul, Republic of Korea; 7 Division of Social Work, Samsung Medical Center, Seoul, Republic of Korea; 8 Department of Mental Health, Johns Hopkins Bloomberg School of Public Health, Baltimore, MD, United States; 9 Cancer Education Center, Samsung Medical Center, Seoul, Republic of Korea

**Keywords:** digital health literacy, digital health, health literacy, surveys and questionnaires, digital inclusion, DHL, general population, national survey, young adult, older adult, cross-sectional survey, cross-sectional, Korea, online survey, face-to-face interview, digital divide, digital health technology

## Abstract

**Background:**

As the rapid advancement of digital health technologies has significantly improved health management, digital health literacy (DHL) has emerged as a crucial determinant of health outcomes.

**Objective:**

The aim of this study was to assess DHL among Korean adults using a task-based assessment tool and to examine how sociodemographic and health literacy factors are associated with DHL, with the goal of identifying vulnerable groups and informing equitable digital health strategies.

**Methods:**

This cross-sectional survey was conducted from July 20 to July 31, 2022, among adults aged 19 years and older in South Korea. Participants were recruited nationally, with those younger than 55 years completing an online survey and those aged 55 years and older participating in face-to-face interviews to address the digital divide. Participants’ DHL was assessed using the 34-item Digital Health Technology Literacy Assessment Questionnaire, a tool designed to evaluate practical task-based competencies.

**Results:**

Among 1041 Korean adults, 27.8% (n=289) demonstrated low DHL, with the largest gaps observed in mobile app use and critical evaluation of health information. The mean DHL score (on a 100-point scale) was 73.8 (SD 29.7). The high and low DHL groups scored 90.3 (SD 35.6) and 31.5 (SD 20.0), respectively. Specifically, only 49 (17%) of the low DHL group were able to sign up for an app, compared with 716 (95.2%) in the high DHL group. Similarly, 52 (18%) of the low DHL group could update an app, whereas 712 (94.7%) could in the high DHL group. Multivariate analysis revealed that older age (≥60 years), lower income (≤US $2000/month), unemployment, and inadequate general health literacy were significantly associated with lower DHL, highlighting the need to target modifiable factors beyond age.

**Conclusions:**

This study highlights the ongoing digital health disparities, emphasizing the need for tailored strategies in mobile apps and digital health technologies. Addressing modifiable factors such as health literacy is key to ensuring equitable access and the effective use of digital health resources.

## Introduction

The rapid advancement of digital health technologies has significantly improved health management, making health care services more efficient and personalized [1,2]. Innovations such as telehealth, electronic health records, wearable fitness trackers, mobile health apps, and digital therapeutics empower individuals to actively manage their health and make informed decisions [3,4]. During the COVID-19 pandemic, digital health technologies played a crucial role in minimizing disruptions to health care delivery, highlighting their importance in future public health crises [5]. Consequently, digital health care has become vital at the national level owing to its potential to enhance health care delivery and outcomes [6]. Health literacy is increasingly recognized as a foundational skill that enables individuals to effectively engage with digital health tools and services. Prior studies have shown that individuals with higher levels of both general health literacy and digital health literacy (DHL) are more likely to adopt digital health technologies, seek reliable online health information, and practice informed self-management [7].

In this context, DHL has emerged as a crucial determinant of health outcomes [4,8]. Inadequate DHL exacerbates critical societal challenges, such as rising health care expenditures, low life expectancy, and decreased ability to access and utilize health services, thereby negatively impacting overall public health [9]. A recent meta-analysis found that interventions designed to improve DHL led to significant improvements in digital skills, health status, and self-management among older adults [10]. DHL encompasses a broader scope of health literacy and refers to individuals’ ability to obtain, process, communicate, and comprehend health information and services, make effective health decisions, and improve health data in the context of using digital health technologies [11]. Therefore, assessing DHL at the national level is necessary to identify the vulnerable populations possibly facing difficulties in accessing, adopting, and utilizing digital health care and technologies [12].

However, studies have measured DHL in the general population using limited measures. Most previous studies have been conducted on specific groups such as young university students or older adults [11,13-15]. A previous study included all age groups; however, it was conducted online using a social networking service and did not sufficiently capture DHL in the general population [16]. Most studies have assessed internet use capabilities for DHL using measures such as the eHealth Literacy Scale [17]. Furthermore, recent instruments such as the Digital Health Literacy Instrument [18], the eHealth Literacy Questionnaire [19], and the Digital Health Literacy Assessment [20] do not assess DHL in the context of the use of digital health technologies.

Recently, the Digital Health Technology Literacy Assessment Questionnaire (DHTL-AQ) was developed to address the limitations of widely used tools such as the Digital Health Literacy Instrument and eHealth Literacy Scale, which primarily assess perceived digital competence. By contrast, the DHTL-AQ directly evaluates users’ ability to perform core digital health tasks—such as navigating health apps, logging in, updating apps, and interpreting app-based information—offering a more objective and practical measure of DHL. This task-based approach is particularly relevant in today’s mobile health environment, where the ability to perform specific digital actions is essential for effective health engagement. The DHTL-AQ demonstrated high internal consistency (Cronbach α=0.92) in a previous validation study among Korean adults [21].

This study aimed to evaluate DHL in a nationally representative sample of Korean adults using the validated DHTL-AQ. We also examined the associations between sociodemographic characteristics, general health literacy, and DHL, with the goal of identifying vulnerable subgroups and informing the development of targeted digital health interventions.

## Methods

### Ethical Considerations

This study was approved by the Institutional Review Board of Samsung Medical Center (IRB No. 2022-02-013-005). All participants provided informed consent before participation. No identifiable personal data were collected, and all responses were anonymized. Participants received a small gift voucher (approximately US $2) as a token of appreciation.

### Participants

We conducted a cross-sectional online survey in South Korea from July 20, 2022, to July 31, 2022. Participants were recruited from a closed national survey panel, which consists of preregistered adult members stratified by region, age, and sex based on national census data. Panel members voluntarily accessed the survey platform and participated on a self-selected basis during the study period. A total of 1041 respondents completed the survey. This stratified quota sampling approach was designed to enhance representativeness of the general adult population in South Korea. Further details on the sampling frame and recruitment procedures have been previously described [22]. The survey platform managed data access and storage.

The inclusion criteria were as follows: (1) adults aged 19 years and older; (2) individuals able to communicate, read, and write independently; and (3) individuals aged 70 years and older based on their cognitive function and capacity to complete the questionnaire. Individuals with central nervous system disorders or a severe neurological or psychiatric history were excluded.

This was a closed survey using a national panel system. Participants younger than 55 years were recruited through a web-based platform, while those aged 55 years and older were recruited via offline, quota-based sampling. Face-to-face interviews were conducted by trained interviewers at participants’ homes or community centers. Older participants were encouraged to complete the survey independently but were offered assistance with difficult items upon request. Because the online survey system required responses to all items before submission, the final dataset contained no missing values. The online survey was conducted in accordance with key elements of the CHERRIES (Checklist for Reporting Results of Internet E-Surveys) checklist for reporting internet-based surveys [23] (see Checklist 1).

### Measures

To assess DHL, we utilized the full version of the DHTL-AQ, consisting of 34 items. This tool was developed and validated in 2020 among Korean adults through a multiphase process that included expert panel review, cognitive interviews, and pilot testing with participants across diverse age groups, including those aged 55 years and older [21]. The DHTL-AQ is a self-reported measure that includes two domains corresponding to four categories: “information and communication technology (ICT) terms,” “ICT icons,” and “use of an app” in the digital functional literacy domain and “evaluating reliability and relevance of health information” in the digital critical literacy domain. The total DHL score was calculated by summing the scores for the 34 items. All items of the DHTL-AQ were measured dichotomously. The degree of self-assessed agreement regarding one’s ability to perform tasks and their knowledge of actual skills was assessed, with each item scored as 1 or 0. ICT terms (11 items) and ICT icons (9 items) were scored as 1 for knowing and 0 for not knowing. Use of an app (9 items) was scored as 1 for being able and 0 for not being able or not knowing. Evaluating the reliability and relevance of health information (5 items) was assessed as 1 for agreeing and 0 for not agreeing, resulting in a range of 0-34.

Participants were divided into two groups based on the DHTL-AQ cutoff value established in the original validation study: the low DHL group (scores <22 of 34) and high DHL group (scores ≥22) [21]. To facilitate a meaningful comparison of competencies across these domains, all scores were converted to a standardized 100-point scale.

Health literacy was measured using the Newest Vital Sign [24], an objective instrument based on six questions about the nutrition label on a container of ice cream; the instrument was validated in Korean [25]. Additionally, these scores (total scores ranging from 0 to 6) were divided into dichotomous variables to classify individuals into three categories: inadequate literacy (0‐1), possibly limited literacy (2-3), and almost always indicating adequate literacy (4–6) levels. Participants were also asked about their sociodemographic characteristics, including sex, age, education level, marital status, current employment status, and monthly household income.

### Statistical Analysis

Descriptive statistics were used to summarize participant characteristics. To compare the low and high DHL groups, we employed independent *t* tests for continuous variables and *χ*^2^ tests for categorical variables, after confirming that the underlying assumptions were satisfied. A Poisson regression model with robust variance estimation was used to examine associations between participant characteristics and levels of DHL. Prevalence ratios (PRs) with 95% CIs were calculated. We adjusted for sex, age, occupational status, monthly household income, and health literacy. All statistical analyses were performed using Stata version 14 (StataCorp LP) and R 4.2.3 (R Foundation for Statistical Computing). Statistical significance was set at *P*<.05, and 2-tailed *P* values were calculated.

## Results

A total of 1041 individuals participated in and completed the survey. The mean age of the participants was 47.1 (SD 16.3) years; 50.3% (n=523) were male. In total, 752 (72.2%) participants had high DHL levels, and 289 (27.8%) had low DHL levels (Table 1). Participants with low DHL levels were more likely to be older and have a lower educational level (*P*<.001). For economic characteristics, participants who were unemployed or had a monthly family income of <US $2000 were more likely to have low DHL levels (Table 1). Moreover, the low DHL group had the highest proportion of participants with inadequate and limited health literacy (Multimedia Appendix 1).

**Table 1.** Comparison of participant characteristics by digital health literacy level.

	Overall (N=1041)		Digital health literacy^a^				
			High (n=752, 72.2%)		Low (n=289, 27.8%)		*P* value^b^
Age (y), mean (SD)	47.1 (16.3)		41.3 (5.5)		62.0 (15.5)		<.001
Median (IQR)	47.0 (34.0‐61.0)		42.0 (30.0‐52.0)		65.0 (53.0‐73.0)		
19-39, n (%)	369 (35.4)		330 (43.9)		39 (13.5)		
40-59, n (%)	422 (40.5)		367 (48.8)		55 (19.0)		
≥60, n (%)	250 (24.0)		55 (7.3)		195 (67.5)		
Sex (male), n (%)	524 (50.3)		379 (50.4)		145 (50.2)		.95
Education, n (%)							<.001
≤Middle school graduation	131 (12.6)		1 (0.1)		130 (45.0)		
High school graduation	323 (31.0)		217 (28.9)		106 (36.7)		
College or university graduation	498 (47.8)		449 (59.7)		49 (17.0)		
Postgraduate or higher	89 (8.5)		85 (11.3)		4 (1.4)		
Marital status, n (%)							<.001
Single	357 (34.3)		318 (42.3)		39 (13.5)		
Married	609 (58.5)		406 (54)		203 (70.2)		
Divorced or widowed	75 (7.2)		28 (3.7)		47 (16.3)		
Occupational status (yes), n (%)	716 (68.8)		555 (73.8)		161 (55.7)		<.001
Monthly household income, n (%)							<.001
≤US $2000	159 (15.3)		54 (7.2)		105 (36.3)		
>US $2000, ≤US $4000	293 (28.1)		203 (27.0)		90 (31.1)		
>US $4000	400 (38.4)		350 (46.5)		50 (17.3)		
Unknown	189 (18.2)		145 (19.3)		44 (15.2)		
Health literacy, n (%)^c^							<.001
Inadequate literacy	126 (12.1)		42 (5.6)		84 (29.1)		
Limited literacy	258 (24.8)		136 (18.1)		122 (42.2)		
Adequate literacy	657 (63.1)		574 (76.3)		83 (28.7)		

a Digital Health Technology Literacy Assessment Questionnaire (low <22, high ≥22, total 34 points).

b *P* values were calculated using independent *t* tests for continuous variables and χ2 tests for categorical variables.

c Health literacy was measured using the Newest Vital Sign (scores range from 0 to 6) to identify persons with inadequate (0-1), limited (2-3), or adequate (4-6) levels of health literacy.

**Figure 1.**
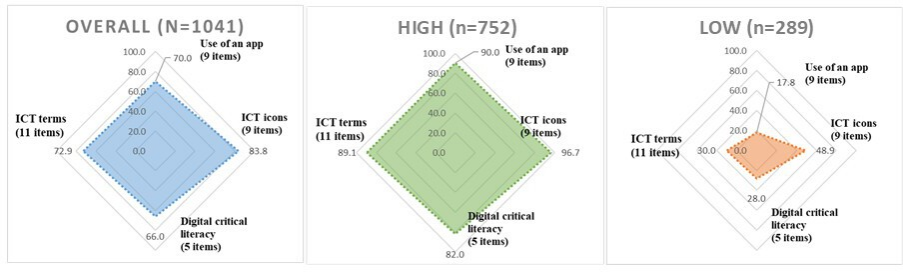
Different digital health literacy in four domains (N=1041). *Digital health literacy measured by the Digital Health Technology Literacy Assessment Questionnaire (low <22, high ≥22, total 34 points). The scores were converted to a scale of 100 for comparison across domains. ICT: information and communication technology.

Figure 1 shows the overall assessment of DHL across a broad sample and compares the domain-specific competencies of the DHLT-AQ between the high (n=752) and low (n=289) DHL groups. We adjusted the total score of 34 points to a 100-point scale to facilitate comparison across domains and found that the mean score of the DHTL-AQ was 73.8 (SD 29.7), with the low and high DHL groups scoring 31.5 (SD 20.0) and 90.3 (SD 35.6), respectively. For the *z* scores, a value of 1.0 signified that the score fell within the top 16% of the distribution. This transformation provided a clear distinction to identify high-scoring groups based on their deviations from the mean. In the overall analysis, the participants displayed a well-distributed DHL, with the highest average score observed for ICT icons (83.8, SD 30.0), followed by ICT terms (72.9, SD 33.6), app usage (70.0, SD 37.8), and digital critical literacy (66.0, SD 38.0). The high DHL group displayed significantly higher competencies across all categories, with the highest scores observed in ICT icons (96.7, SD 11.1) and the use of an app (90.0, SD 17.8), followed by ICT terms (89.1, SD 19.1) and digital critical literacy (82.0, SD 28.0). By contrast, the low DHL group reported significantly lower competencies, particularly in app usage (17.8, SD 25.6) and digital critical literacy (28.0, SD 34.0). Across all four domains, the low DHL group consistently showed lower competencies than the high DHL group, with scores ranging from 17.8 to 48.9 (Figure 1).

An analysis of individual items within each domain revealed that a significant proportion of participants in the high DHL group were proficient in app use (Table 2). For example, 84.8% (n=638) of the high DHL group could easily find a health-related app compared with 19.4% (n=56) of the low DHL group. Similarly, 95.2% (n=716) and 94.7% (n=712) of the high DHL group could sign up for and update an app compared with 17% (n=49) and 18% (n=52) of the low DHL group, respectively.

**Table 2.** Digital health literacy of the general population by item.

	Overall		Digital health literacy^a^			
	(N=1041), n (%)		High (n=752), n (%)		Low (n=289), n (%)	
Use of an app (9 items), yes						
I can easily find the app to help my health.	694 (66.7)		638 (84.8)		56 (19.4)	
I can find more reliable apps by comparing different apps.	629 (60.4)		583 (77.5)		46 (15.9)	
I can sign up to use the app (create ID, password, etc).	765 (73.5)		716 (95.2)		49 (17.0)	
I can update the app.	764 (73.4)		712 (94.7)		52 (18.0)	
I can record my health information through the app.	717 (68.9)		672 (89.4)		45 (15.2)	
I can use the recorded health information for my health through the app.	702 (67.4)		658 (87.5)		44 (16.3)	
I can record the amount of activity (steps), weight, and meals through the app.	741 (71.2)		694 (92.3)		47 (17.6)	
I can check the amount of activity (steps), weight, and meals recorded through the app.	746 (71.7)		695 (92.4)		51 (22.5)	
I can set preferences (sound, security, display, notification, etc) for the app.	780 (74.9)		715 (95.1)		65 (22.5)	
ICT^b^ icons (9 items), know						
Icon (URL)	828 (79.5)		712 (94.7)		116 (40.1)	
Icon (search bar)	854 (82.0)		714 (94.9)		140 (48.4)	
Icon (social media)	848 (81.5)		736 (97.9)		112 (38.8)	
Icon (bluetooth)	860 (82.6)		726 (96.5)		134 (46.4)	
Icon (download)	859 (82.5)		727 (96.7)		132 (45.7)	
Icon (synchronization)	826 (79.3)		720 (95.7)		106 (36.7)	
Icon (voice assistant)	896 (86.1)		739 (98.3)		157 (54.3)	
Icon (security file)	885 (85.0)		738 (98.1)		147 (50.9)	
Icon (QR code)	957 (91.9)		738 (98.1)		219 (75.8)	
ICT terms (11 items), know						
Play Store, App Store	833 (80.0)		724 (96.3)		109 (37.7)	
Bluetooth	873 (83.9)		732 (97.3)		141 (48.8)	
Wearable device	785 (75.4)		693 (92.2)		92 (31.8)	
Application	737 (70.8)		648 (86.2)		89 (30.8)	
Web browser	851 (81.7)		708 (94.1)		143 (49.5)	
Cloud	676 (64.9)		640 (85.1)		36 (12.5)	
Update, synchronization	915 (87.9)		724 (96.3)		191 (66.1)	
QR code	686 (65.9)		646 (85.9)		40 (13.8)	
Domain, URL	598 (57.4)		559 (74.3)		39 (13.5)	
Search bar	699 (67.1)		655 (87.1)		44 (15.2)	
Chatbot, voice assistant	700 (67.2)		662 (88.0)		38 (13.1)	
Digital critical literacy (5 items), yes						
I can judge whether the information I got from the internet or digital health is reliable.	693 (66.6)		598 (79.5)		95 (32.9)	
I can judge whether the information on the internet or digital health was used for commercial benefit.	625 (60.0)		556 (73.9)		69 (23.9)	
I can check whether the same information is provided by other websites or on the internet.	707 (67.9)		627 (83.4)		80 (27.7)	
I can judge whether the information I find on the internet or in digital health is properly used for myself.	701 (67.3)		624 (83.0)		77 (26.6)	
I can use the information I find on the internet or digital health to make health-related decisions.	743 (71.4)		654 (87.0)		89 (30.8)	

a Digital health literacy measured by the Digital Health Technology Literacy Assessment Questionnaire (low <22, high ≥22, total 34 points).

b ICT: information and communication technology.

The most pronounced disparities in ICT icon recognition were observed for the URL icon, identified by 712 of 752 (94.7%) participants in the high DHL group compared with 116 of 289 (40.1%) in the low DHL group, and for the synchronization icon, recognized by 720 of 752 (95.7%) participants versus 106 of 289 (36.7%) participants, respectively (Table 2).

For digital critical literacy, 598 (79.5%) participants of the high DHL group reported being able to judge the reliability of online health information compared with 95 (32.9%) participants of the low DHL group. Confidence in using online health information was also higher in the high DHL group (n=654, 87% vs n=89, 30.8%; Table 2).

Younger age, higher income, and better health literacy were independently associated with higher DHL. Individuals aged 19‐39 years had a PR of 2.53 (95% CI 2.19‐2.91), and those aged 40‐59 years had a PR of 2.43 (95% CI 2.11‐2.80), both compared with participants aged 60 years and older (Table 3). Compared with individuals with a monthly household income of ≤US $2000, those earning >US $2000 to ≤US $4000 had a PR of 1.19 (95% CI 1.07‐1.33), and those earning >US $4000 had a PR of 1.30 (95% CI 1.17‐1.44). For health literacy, participants with limited literacy had a PR of 1.31 (95% CI 1.14‐1.33), and those with adequate literacy had a PR of 1.60 (95% CI 1.42‐1.82), both relative to the inadequate literacy group.

**Table 3.** Factors associated with appropriate digital health literacy.^a^

	Digital health literacy			
	Crude model, PR^b^ (95% CI)		Adjusted model, PR (95% CI)	
Sex, female	1.00 (0.93-1.08)		1.03 (0.98-1.09)	
Age (years), mean (SD)				
19‐39	4.07 (3.33-4.96)		2.53 (2.19-2.91)	
40‐59	3.95 (3.24-4.83)		2.43 (2.11-2.80)	
≥60	Reference		Reference	
Education, ≥college or university graduation	1.89 (1.74-2.06)		1.00 (0.96-1.04)	
Marital status				
Single, divorced, or widowed	1.20 (1.12-1.29)		1.00 (0.93-1.08)	
Married	Reference		Reference	
Occupational status (yes)	1.28 (1.17-1.40)		1.09 (1.02-1.16)	
Monthly household income				
≤US $2000	Reference		Reference	
>US $2000, ≤US $4000	2.04 (1.67-2.49)		1.19 (1.07-1.33)	
>US $4000	2.58 (2.12-3.13)		1.30 (1.17-1.44)	
Unknown	2.23 (1.84-2.77)		1.24 (1.11-1.39)	
Health literacy^c^				
Inadequate literacy	Reference		Reference	
Limited literacy	1.58 (1.26-2.49)		1.31 (1.14-1.33)	
Adequate literacy	2.62 (2.12-3.24)		1.60 (1.42-1.82)	

a Adjusted for sex, age, occupational status, monthly household income, and health literacy.

b PR: prevalence ratio.

c Health literacy was measured using the Newest Vital Sign (scores range from 0 to 6) to identify persons having inadequate (0‐1), limited (2-3), or adequate (4-6) levels of health literacy.

## Discussion

This study evaluated DHL among a nationally representative sample of Korean adults using the DHTL-AQ, a task-based instrument designed to capture functional and critical digital competencies. Our findings revealed that 27.8% of the population demonstrated low levels of DHL, with the most pronounced difficulties observed in mobile health app usage. Furthermore, lower DHL was significantly associated with older age, unemployment, lower income, and inadequate health literacy, indicating persistent inequalities in digital engagement.

Our study provides a comprehensive evaluation of DHL across the general population and offers insights into the challenges faced by people using digital health technologies. Notably, individuals in the low DHL group, approximately one-third of the participants, struggled with mobile health app usage. This is concerning as health care increasingly relies on mobile technologies for patient care. The most vulnerable populations such as older adults and those with chronic conditions are often the least equipped with the necessary digital literacy skills. Lower DHL among older individuals may stem from a generational gap, primarily characterized by limited exposure to rapidly evolving digital environments. This pattern is not unique to Korea; similar trends have been observed globally. A recent scoping review on digital engagement among older adults across various countries, including those in Europe and North America, reported consistent barriers related to age, digital familiarity, and technology access [26]. To bridge this digital divide, developing tailored educational programs and increasing exposure to digital technologies in these vulnerable populations are crucial. These findings are also in line with Korean studies demonstrating that targeted education and repeated exposure can effectively enhance digital engagement in these groups [27-29].

In this study, we conducted a detailed survey on mobile app usage, specifically based on features commonly used in apps by individuals with chronic diseases, unlike previous studies that focused solely on internet-based health information [17]. We assessed the broader knowledge and skills necessary for the effective use of digital health technologies, such as ICT-related icons, terminology, and mobile app tasks [20]. The survey focused on the key aspects of mobile health app use, including finding, comparing, and selecting apps; logging in and updating apps; and recording, using, and checking health information. We found that most participants in the low DHL group faced challenges in comparing apps to find reliable ones. These results are consistent with the existing literature, indicating that less awareness of apps and difficulties finding and using health apps are major challenges among digitally excluded populations [30]. Therefore, helping users with low DHL identify which of the many apps are trustworthy and easy to use for their health is essential, including training them in evaluating and properly searching for apps [31,32]. Few studies have reported on the functionality of mobile device usage. In a Chinese study in 2022, 62.55% of adults had a mobile app, and 27.61% reported the ability to install apps by themselves [33]. A study on older adults in Korea found that 63.2% of participants could install or delete apps on their smartphones on their own and that their partners and children were their main supporters [34]. Recent increases in mobile apps have significantly enhanced the effectiveness of self-care management and facilitated communication, education, recording, and use of data to promote healthy outcomes [35,36]. Additionally, previous review studies have confirmed that interventions using mobile health apps may enhance the quality of life and self-efficacy and reduce anxiety, depression, and distress [37,38]. Therefore, this task-based approach offers a more realistic understanding of digital health competencies by directly evaluating tasks such as logging into and updating apps. Beyond individual skills, our findings suggest broader implications for the integration of digital health into health care systems. As tools like mobile health apps, electronic patient portals, and telehealth services become embedded in routine care, gaps in DHL may limit patients’ ability to access essential services, communicate effectively with providers, and engage in self-management. These barriers may undermine the full potential of digital health innovations unless systems are designed to be inclusive and literacy-sensitive. To promote equitable access, health care systems must prioritize the development of user-friendly technologies, offer training tailored to different literacy levels, and integrate digital health literacy support into care delivery.

Regarding ICT-related icons and terms, individuals with low DHL were unfamiliar with terms like “cloud,” “QR code,” “domain,” “search bar,” and “chatbot.” Literacy in any domain, including DHL, relies on the ability to understand its unique vocabulary. However, participants who did not recognize the term “QR code” were able to understand it through the associated icon. This suggests that using icons can be an effective way to improve DHL, especially in individuals with low DHL. Prior research supports this approach because it simplifies the learning process and builds on existing knowledge to encourage engagement with digital health technologies [39].

Despite the significant differences in overall competencies, both the high and low DHL groups scored low in evaluating the reliability and relevance of health information, indicating a shared deficiency in digital critical literacy. Our findings align with those of previous studies and highlight the need for improved digital competence, particularly in the assessment and effective use of digital health information [40,41]. Although competencies related to evaluating the relevance and trustworthiness of health information are important, this study highlights the broader need to expand the dissemination of critical literacy within the digital health ecosystem [4]. This deficiency can lead to the spread of health misinformation and increase the risk of inappropriate health behavior [42]. Vulnerable groups may also struggle with a lack of personal interaction with digital health services, limiting their ability to fully engage in health promotion and risk prevention. Therefore, health care organizations must regularly assess DHL and address these competency gaps through targeted interventions [42].

Furthermore, our results revealed that older age, unemployment, and lower household income were significantly and independently associated with lower DHL. As previously mentioned, young adults are often viewed as a demographic with extensive connectivity, whereas older adults are seen as a susceptible segment that experiences digital inequality because of ageism [43]. While previous studies [31] identified sex and age as significant factors influencing eHealth literacy related to app usage among Seoul residents, our findings suggest that greater attention should be paid to socioeconomic factors, including occupation and income. In a recent cross-sectional study conducted in Korea with older adults, 87.1% of the participants were mobile app users, and 57.4% reported using health care apps [34]. This study confirmed that age cannot be considered an impenetrable barrier to improving DHL. One study found that older adults hold positive attitudes toward acquiring health information from the internet and have comparable eHealth literacy with young adults in education [44]. Therefore, the age-related disparity is expected to diminish within the next decade, as recent studies suggest that differences between the two age groups may virtually disappear when the current middle-aged individuals enter the oldest age group [45]. This trend suggests a growing opportunity to address digital inequality through interventions that promote digital familiarity and engagement, particularly among older adults.

We also demonstrated that lower health literacy was significantly associated with lower DHL. Limited health literacy can be considered a barrier to digital health implementation; therefore, addressing low health literacy was required to address DHL [46]. This was consistent with the results of this study, which indicated that people with adequate health literacy are more likely to have 1.6 times higher DHL than those with inadequate health literacy. Although previous studies have suggested that self-reported health literacy may not directly influence digital technology use, individuals with low health literacy often rely on traditional communication channels, such as SMS text messages and radio, for health information [47]. These findings suggest that improving health literacy may be an essential foundation for narrowing digital health gaps, particularly among vulnerable populations.

Taken together, the magnitude of these associations—such as a 2.5-fold higher likelihood of high DHL among younger adults and a 60% increase among those with adequate literacy—highlights the practical significance of sociodemographic and literacy disparities. These findings underscore the importance of tailoring interventions for digitally vulnerable groups to promote equitable engagement with digital health technologies. Although South Korea is a highly digitalized society with near-universal smartphone use and robust internet infrastructure, our findings reveal persistent gaps in DHL. This suggests that in low-income countries—where access to technology, education, and digital health infrastructure is more limited—DHL disparities may be even more pronounced. Therefore, foundational investments in digital access, health education, and user-centered design are critical in global efforts to promote equitable digital health engagement.

This study has several limitations. First, although stratified quota sampling based on regional and sociodemographic variables was used to reflect the Korean adult population, the final sample may still be subject to selection bias. Specifically, individuals with very limited digital access or those residing in remote rural areas may have been underrepresented. Second, although we adopted a hybrid survey mode (online for participants younger than 55 years and in-person for those aged 55 years and older) to mitigate digital exclusion, the possibility remains that survey respondents were more digitally capable than the general population. Third, the cross-sectional design limits causal inference. Fourth, the DHL instrument relied in part on self-reported measures, which may introduce response bias; however, the inclusion of task-based items improved measurement robustness. While the online survey system ensured complete data with no missing values, this design may have introduced potential biases. Specifically, the forced-response format may have led some participants to provide nonreflective or arbitrary answers on items about which they were uncertain. Finally, while we adjusted for key sociodemographic factors, unmeasured variables—such as prior experience with digital tools, health status, regional digital infrastructure, and local health service access—may also influence DHL. Future studies should incorporate these contextual and structural variables to better understand the determinants of DHL.

In conclusion, this study found that nearly one-third of Korean adults exhibit low DHL, with older age, lower income, unemployment, and limited health literacy being significant correlates. These findings highlight the persistent nature of digital inequalities, even in a highly digitalized society. To address these gaps, targeted interventions that incorporate user-friendly mobile health technologies and improve general health literacy are essential. Our findings have important implications for advancing equitable digital health strategies not only in Korea but also in other global contexts. Although South Korea is a highly digitalized society with near-universal smartphone use and robust internet infrastructure, our findings reveal persistent gaps in DHL. This suggests that in low-income countries—where access to technology, education, and digital health infrastructure is more limited—DHL disparities may be even more pronounced. Therefore, foundational investments in digital access, health education, and user-centered design are critical in global efforts to promote equitable digital health engagement.

## Supplementary material

10.2196/67780 Multimedia Appendix 1 Distribution of health literacy by digital health literacy level.

10.2196/67780 Checklist 1 CHERRIES checklist.

## Abbreviations

CHERRIES: Checklist for Reporting Results of Internet E-Surveys
DHL: digital health literacy
DHTL-AQ: Digital Health Technology Literacy Assessment Questionnaire
ICT: information and communication technology
PR: prevalence ratio
