# Supplementary figures and images for: Digital Health Literacy in the General Population: National Cross-Sectional Survey Study
Source: J Med Internet Res. 2025 Sep 22;27:e67780. doi: 10.2196/67780 (PMC12453288; doi:10.2196/67780)

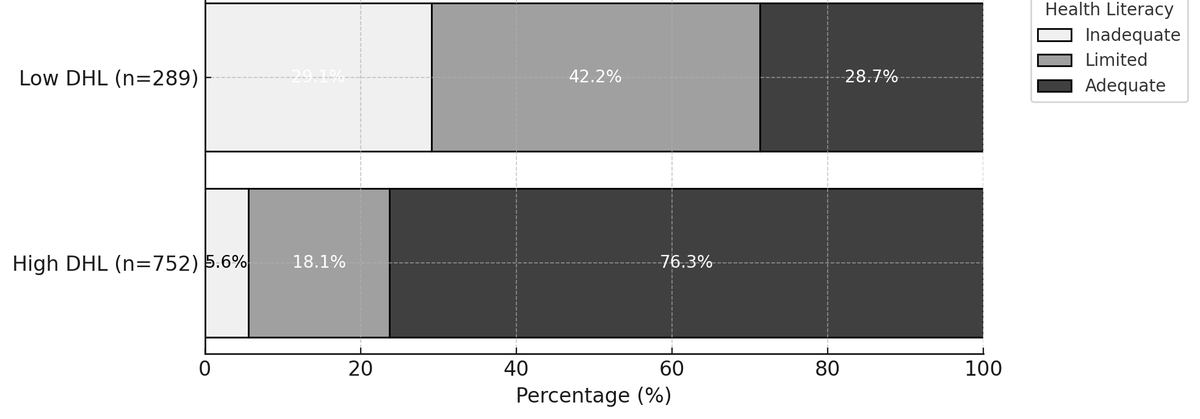

Supplement: Multimedia Appendix 1 [file jmir-v27-e67780-s001.png]
